# Increased Serum ANGPTL8 Concentrations in Patients with Prediabetes and Type 2 Diabetes

**DOI:** 10.1155/2017/8293207

**Published:** 2017-09-07

**Authors:** Yanhua Yin, Xiaoying Ding, Liang Peng, Yanqiang Hou, Yunxia Ling, Mingyu Gu, Yufan Wang, Yongde Peng, Haiyan Sun

**Affiliations:** ^1^Department of Endocrinology and Metabolism, Shanghai General Hospital, Shanghai Jiao Tong University School of Medicine, 100 Haining Rd, Shanghai 200080, China; ^2^Department of Clinical Laboratory Center, Shanghai Songjiang District Central Hospital, Shanghai 200080, China

## Abstract

The objectives of the study were to investigate serum ANGPTL8 concentrations in different glucose metabolic statuses and to explore the correlations between serum ANGPTL8 levels and various metabolic parameters. Serum ANGPTL8 levels were determined using ELISA in 22 subjects with NGT (normal glucose tolerance), 74 subjects with IGR (impaired glucose regulation), and 33 subjects with T2DM (type 2 diabetes mellitus). Subjects with IFG, IGT, CGI, and T2DM had higher levels of serum ANGPTL8 than subjects with NGT. Serum ANGPTL8 was positively correlated with FPG, fasting C-peptide, and postprandial C-peptide and negatively correlated with BETA/IR when adjusted for age and BMI. Multivariate analysis suggested FPG and fasting C-peptide as independent factors associated with serum ANGPTL8 levels. Serum ANGPTL8 concentrations were significantly increased in IGR and T2DM. Serum ANGPTL8 might play a role in the pathological mechanism of glucose intolerance.

## 1. Introduction

ANGPTL8 (angiopoietin-like protein 8), also called TD26, RIFL (refeeding-induced fat and liver), lipasin, betatrophin, and C19orf80 (chromosome 19 open reading frame 80), is a novel protein that is primarily expressed in the liver and fat. ANGPTL8 plays a role in regulating lipid metabolism in mice and in vitro tests. Ren et al. showed that RIFL-null mice had lower serum triglyceride levels than the wild-type mice [1]. In Quagliarini et al.'s study, plasma TG (triglyceride) level did not change in mice expressing ANGPTL3 alone, whereas coexpression with ANGPTL8 resulted in hypertriglyceridemia, despite a reduction in circulating ANGPTL3 [2]. Zhang reported that obesity increases liver lipasin, whereas fasting reduces its expression in fat. Lipasin overexpression in mice increases serum triglyceride levels [3]. Despite its role in regulating lipid metabolism, whether ANGPTL8 is related to glucose metabolism was controversial. Elevated circulating ANGPTL8 levels were found in subjects with DM in Espes et al.'s reports [4–6], while in Gómez-Ambrosi et al.'s, ANGPTL8 levels declined [7, 8].

To address this question, the study evaluated the association of ANGPTL8 with different glucose metabolic statuses, including IFG (isolated impaired fasting glucose), IGT (isolated impaired glucose tolerance), and CGI (combined glucose intolerance). We demonstrated a significantly elevated serum ANGPTL8 level in IGR subjects, suggesting that ANGPTL8 might play a role before developing into diabetes mellitus.

## 2. Materials and Methods

### 2.1. Study Subjects

**Figure 1:**
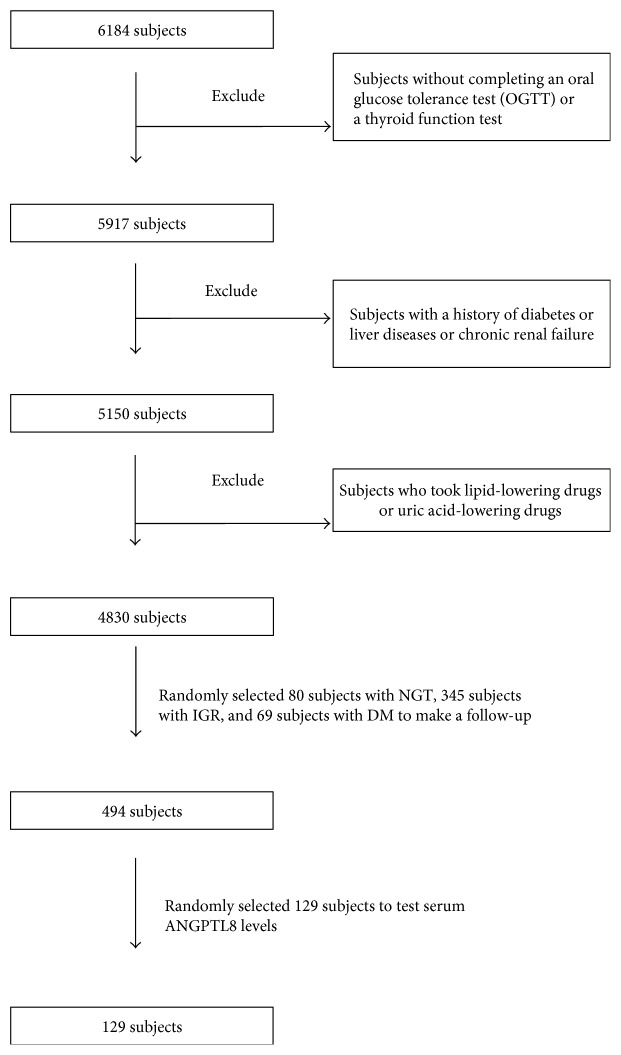
Process of exclusion and inclusion.

This study was part of an epidemiologic study of diabetes, thyroid disease, and osteoporosis, which took place in the countryside of Sijing, Shanghai, China. From July 2012 to March 2013, 6184 subjects participated in the survey. We made a follow-up in 494 subjects from October to November in 2014. The 129 subjects which were randomly selected including 22 subjects with NGT, 74 subjects with IGR (30 with IFG, 32 with IGT, and 12 with CGI), and 33 subjects with T2DM (type 2 diabetes mellitus) were included to examine serum ANGPTL8 levels. The diagnosis of T2DM and prediabetes was based on the criteria in the American Diabetes Association's 2003 guidelines. None had gestational diabetes, active hepatitis/liver cirrhosis, chronic renal failure on hemodialysis, congestive heart failure, or other known major diseases. Subjects who were treated with lipid-lowering drugs or uric acid-lowering drugs were also excluded to avoid the possible confounding effects of medications (Figure 1()). The study was approved by the medical ethics committee of Shanghai General Hospital. All participants gave written informed consent to participate in this research.

### 2.2. Anthropometric and Biochemical Measurements

Blood samples were collected after at least 10 h of overnight fasting. Participants with no history of diabetes were given a standard 75 g glucose solution, whereas for safety reasons, participants with T2DM were given a steamed bun that contained approximately 80 g of complex carbohydrates. Blood samples were drawn zero, and 120 min after, the glucose or carbohydrate load was ingested to measure glucose concentrations. Plasma glucose, cholesterol (TC), triglyceride (TG), high-density lipoprotein cholesterol (HDL-C), and low-density lipoprotein cholesterol (LDL-C) levels were assessed enzymatically by an automatic biochemistry (HITACHI 7600) analyzer with WOKO reagent (SANWA INTL CO.). Glycosylated hemoglobin (HbA1c) was detected by high-performance liquid chromatography (Hemoglobin Analyzer D-10; Bio-Rad Laboratories Inc., Shanghai, China). Fasting serum insulin (FINS), postprandial serum insulin (PINS), fasting C-peptide, and postprandial C-peptide were tested using an electrochemiluminescence analyzer (Roche Cobas e170). Blood pressure (BP) was measured three times while the subject sat calmly. Weight (without shoes and any heavy clothing) and height were measured. Waist circumferences were measured at the narrowest point between the lowest rib and the uppermost lateral border of the iliac crest, and the hips were measured at their widest point. Serum ANGPTL8 concentrations were determined using a validated ELISA kit (Phoenix Pharmaceuticals, Phoenix, USA; catalog number EK-051-55) with intra- and interassay coefficients of variation being <10% and <15%, respectively.

### 2.3. Definitions

Systolic BP (SBP) and diastolic BP (DBP) were calculated as the mean of the three measurements. Body mass index (BMI) was calculated using the following formula: weight (kilograms)/height squared (meters squared). The waist-to-hip ratio (WHR) was calculated using the following formula: waist circumferences/hip circumferences. The homoeostasis model assessment of insulin resistance (HOMA-IR) and the homoeostasis model assessment of beta cell function index (HOMA-BETA) were calculated using the following formulas: HOMA-IR = fasting plasma glucose (FPG) (mM) × insulin (mIU/L)/22.5; HOMA-BETA = 20 × insulin/(FPG − 3.5); BETA/IR = HOMA-BETA/HOMA-IR; ΔI_120_/ΔG_120_ = (PINS − FINS)/(PPG − FPG); and ΔI_120_/ΔG_120_/IR = (PINS − FINS)/(PPG − FPG)/HOMA-IR. According to the 2003ADA recommendations for the diagnosis of diabetes, normal glucose tolerance (NGT) was defined as FPG < 5.6 mM and postprandial plasma glucose (PPG) < 7.8 mM; IFG was defined as 5.6 mM ≤ FPG < 7.0 mM and PPG < 7.8 mM; IGT was defined as FPG < 5.6 mM and 7.8 mM ≤ PPG < 11.1 mM; CGI was defined as 5.6 mM ≤ FPG < 7.0 mM and 7.8 mM ≤ PPG < 11.1 mM; and DM was defined as FPG ≥ 7.0 mM or PPG ≥ 11.1 mM.

### 2.4. Statistical Analyses

Statistical analysis was performed using SPSS 21.0. Continuous data with a normal distribution were expressed as the means ± SD, and data with a skewed distribution were expressed as medians (interquartile range). One-way ANOVA was used for the normally distributed data, and the Kruskal-Wallis test was used for the nonnormally distributed data or data with different variances. *χ*^2^ test was used to compare the gender differences among the groups. Pearson's correlation was used for the normally distributed data, and Spearman's correlation was used for the nonnormally distributed data to examine the association among variables. Partial correlation was used for the adjustment of age and BMI. Multivariate regression analysis was to assess the associations between ANGPTL8 and the variables. All *P* values are two tailed. Significance was determined at *P* < 005.

## 3. Results

### 3.1. Characteristics of Subjects in NGR, IFG, IGT, CGI, and T2DM

There were no gender differences among different glucose metabolic statuses. Subjects with T2DM were significantly older than subjects with NGT. Subjects with CGI had higher levels of BMI, waist circumferences, WHR, FPG, PPG, FINS, PINS, fasting, and postprandial C-peptide, but lower BETA/IR than subjects with NGT. Subjects with IGT had higher levels of WHR, TG, PPG, and PINS than subjects with NGT. Subjects with IFG had higher levels of waist circumferences, WHR, TG, FPG, and HOMA-IR, but lower BETA/IR than subjects with NGT. Serum levels of ANGPTL8 were significantly higher in subjects with IFG, IGT, CGI, and T2DM when compared to subjects with NGT (1.07 ± 0.52 ng/mL, 0.92 ± 0.57 ng/mL, 1.23 ± 0.48 ng/mL, and 0.85 ± 0.67 ng/mL versus 0.38 ± 0.25 ng/mL, *P* < 0.001) (Table 1).

### 3.2. The Relationship between Serum ANGPTL8, Fasting C-peptide, and Metabolic Parameters

We investigated the relationship between serum ANGPTL8 levels and various parameters in all subjects. Serum ANGPTL8 correlated positively with waist circumferences, WHR, FPG, FINS, PINS, fasting C-peptide, postprandial C-peptide, and HOMA-IR, but negatively with BETA/IR. Serum ANGPTL8 still remained positively correlated with FPG, fasting C-peptide, and postprandial C-peptide and negatively correlated with BETA/IR after adjustment for age and BMI (Table 2). Multiple stepwise regression analysis was performed with serum ANGPTL8 as a dependent variable and various parameters including sex, age, BMI, blood pressure, blood lipid, HbA1C, FINS, PINS, fasting C-peptide, and postprandial C-peptide as the independent variables. The result showed that FPG and fasting C-peptide were independently related factors influencing serum ANGPTL8 levels (*β* for FPG = 0.072, *P* = 0.040; *β* for fasting C-peptide = 0.120, *P* = 0.032).

We then investigated the relationship between fasting C-peptide and various parameters in all subjects. Fasting C-peptide correlated positively with waist circumferences, TG, FPG, PPG, FINS, PINS, postprandial C-peptide, HOMA-IR, and HOMA-BETA, but negatively with BETA/IR and ΔI_120_/ΔG_120_. Fasting C-peptide still remained positively correlated with FPG, FINS, PINS, postprandial C-peptide, HOMA-IR, and HOMA-BETA and negatively correlated with BETA/IR after adjustments for age and BMI (Table 2).

Furthermore, we, respectively, investigated the relationship between ANGPTL8 levels and lipids in NGT, IGR, and T2DM, and we did not find any correlations between serum ANGPTL8 levels and lipids (Table 3).

## 4. Discussion

In the recent years, the association of circulating ANGPTL8 concentrations and the hyperglycemic state have become topics of interest. Previous reports have shown controversy in DM. Some showed elevated circulating ANGPTL8 concentrations in subjects with DM in comparison with normal subjects [4–6], while others not. Besides, few had invested the levels of ANGPTL8 in IGR subjects, a high-risk population for T2DM. In this study, we investigated serum ANGPTL8 concentrations in different glucose metabolic statuses including NGT, IFG, IGT, CGI, and T2DM.

IGR, or called prediabetes, is an intermediate state of hyperglycemia with glycemic parameters above normal but below the diabetes threshold [9]. It can be classified into three states: IFG, IGT, and CGI. IGR remains a state of high risk for developing diabetes with yearly conversion rate of 5%–10% [10]. In our study, we found that IFG, IGT, CGI, and T2DM subjects had significantly higher serum ANGPTL8 levels than NGT, but serum ANGPTL8 levels among IGT, CGI, and T2DM had no significant differences. This suggested that ANGPTL8 might act as a predictive marker in prediabetes and diabetes mellitus.

C-peptide is a cleavage protein released during insulin production from its precursor proinsulin. C-peptide is used as a maker to indicate the level of endogenous insulin reserve and *β*-cell function. Our study showed that fasting C-peptide positively correlated with FPG, FINS, PINS, postprandial C-peptide, HOMA-IR, and HOMA-BETA and negatively correlated with BETA/IR after adjustment for age and BMI. This suggested that islet *β*-function increased in insulin-resistant subjects in the early stage of the hyperglycemic state including IGR and newly diagnosed DM. Moreover, we found that after adjustment for age and BMI, serum ANGPTL8 remained positively correlated with FPG, fasting C-peptide, and postprandial C-peptide and negatively correlated with BETA/IR. Taken together, ANGPTL8 levels increased with the level of FPG and increased with the level of fasting C-peptide because of insulin resistance. Serum ANGPTL8 might play a role in the pathological mechanism of glucose intolerance.

Multiple studies have identified that ANGPTL8 plays a role in the regulation of lipid metabolism in mice [2, 3, 11]. Zhang found that lipasin overexpression by adenoviruses in mice increases serum triglyceride levels and a recombinant lipasin inhibits LPL activity [3]. Fu et al. suggested that mice injected with the effective antibody or with lipasin deficiency had increased postprandial cardiac LPL activity and lower TAG levels in the fed state [12]. In addition, Quagliarini et al.'s group observed that ANGPTL8 expression in the livers of mice causes hypertriglyceridemia that is exacerbated by coexpression of ANGPTL3 [2]. But in a human being, the results were controversial. The study performed by Chung et al. suggested that ANGPTL8 concentrations were positively associated with triglycerides (TGs) [13]. A similar result was also observed in Gao et al.'s case-control study [14]. But in Espes et al.'s study, the correlation between ANGPTL8 and TG in patients with T2DM was negative [4].

In our study, we did not find any association between serum ANGPTL8 and lipid profile. This was in accordance with Guo et al.'s study in obese individuals [15] and Hu et al.'s in newly diagnosed type 2 diabetes [6].One reason for the discrepancy as Fu et al. described was the differences in ANGPTL8 antibodies, being against the N-terminus or the C-terminus, in ELISA kits used in different studies [16]. The other reason for the discrepancy may be that the lipid levels in our subjects were concentrated in a small range because of small sample size, not scattered in a larger range that might reflect the real lipid levels in population.

In conclusion, the findings of our cross-sectional study suggested that serum ANGPTL8 might play a role in the hyperglycemic state. However, the exact mechanisms of ANGPTL8 action in the hyperglycemic state have not yet been studied.

The present study had several limitations. First, the study was a cross-sectional design. Although we found elevated serum ANGPTL8 in prediabetes and T2DM and the association between serum ANGPTL8 and other metabolic parameters, it was hard to decide the cause-and-effect relationship between them. Second, our analyses were based on a single detection of blood ANGPTL8. Third, the small sample size of this study might lead to selection bias. We will expand the sample size to further explore the relationship between ANGPLT8 and hyperglycemia in further studies.

## 5. Conclusion

We found that serum ANGPTL8 concentrations were significantly increased in IGR and T2DM. Serum ANGPTL8 were positively correlated with FPG and fasting C-peptide and negatively correlated with BETA/IR after adjustment for sex, age, and BMI. Serum ANGPTL8 might play a role in the pathological mechanism of glucose intolerance.

## Figures and Tables

**Table 1:** Characteristics of 129 subjects, including a statistical comparison of characteristics of subjects with NGR, IFG, IGT, CGI, and T2DM.

	NGT (22)	IFG (30)	IGT (32)	CGI (12)	T2DM (33)	Value	*P*	*P* < 0.05
Gender (M/F)	6/16	17/13	14/18	6/6	21/12	8.026	0.091	
Age (years)	54.41 ± 5.59	57.66 ± 5.50	58.09 ± 6.47	58.17 ± 8.33	60.18 ± 5.86	2.923	0.024	d
BMI (kg/m^2^)	24.21 ± 3.17	25.44 ± 2.63	25.14 ± 3.18	27.20 ± 3.25	26.53 ± 3.36	2.802	0.029	c
Waist circumferences (cm)	79.89 ± 7.29	85.54 ± 7.10	84.25 ± 7.59	89.12 ± 9.16	87.58 ± 7.61	4.432	0.002	a, c, d
WHR	0.85 ± 0.05	0.90 ± 0.04	0.88 ± 0.04	0.91 ± 0.05	0.90 ± 0.04	3.927	0.005	a, b, c, d
SBP (mmHg)	127.00 ± 13.82	128.07 ± 13.55	130.06 ± 14.63	131.67 ± 13.06	132.79 ± 10.62	0.860	0.490	
DBP (mmHg)	80.82 ± 7.24	80.69 ± 5.91	82.12 ± 7.14	83.67 ± 6.25	84.18 ± 7.63	1.359	0.252	
TC (mmol/L)	1.09 (0.77–2.11)	1.95 ± 1.16	2.13 (1.38–3.05)	1.86 (1.20–3.33)	2.02 ± 0.99	7.540	0.110	
TG (mmol/L)	0.44 ± 0.17	0.65 (0.37–1.10)	0.67 (0.47–1.37)	0.65 (0.43–1.01)	0.67 (0.44–1.13)	12.782	0.012	a, b, d
LDL-C (mmol/L)	0.84 ± 0.59	0.92 ± 0.75	1.06 ± 0.69	1.20 ± 0.90	0.98 ± 0.58	0.714	0.584	
HDL-C (mmol/L)	0.53 ± 0.27	0.57 ± 0.38	0.68 ± 0.36	0.71 ± 0.53	0.65 ± 0.36	0.902	0.465	
HbA1C (mmol/L)	5.52 ± 0.31	5.58 ± 0.28	5.67 ± 0.34	5.71 ± 0.33	6.96 ± 1.66	14.084	<0.001	d, g, i, j
FPG (mmol/L)	4.92 ± 0.35	5.96 ± 0.27	5.17 (5.02–5.42)	6.09 (5.75–6.41)	7.40 (6.71–8.55)	97.295	<0.001	a, c, d, e, g, h, i, j
PPG (mmol/L)	6.72 (5.89–6.95)	6.23 ± 1.09	8.50 (8.12–9.80)	9.25 (8.21–9.79)	14.51 ± 4.26	105.764	<0.001	b, c, d, e, f, g, i, j
FINS (mIU/L)	1.80 (1.37–3.62)	3.45 (1.98–4.99)	3.39 (2.46–6.44)	5.67 (2.98–7.59)	4.36 (2.12–8.65)	18.375	0.001	c, d
Fasting C-peptide (ng/mL)	1.18 ± 0.80	1.50 ± 0.60	1.49 ± 0.84	1.64 ± 0.87	1.86 ± 1.18	2.864	0.026	c, d
PINS (mIU/L)	10.72 (6.13–15.20)	17.54 (6.98–34.68)	35.26 (23.84–51.49)	38.60 (23.52–58.64)	21.22 (11.59–39.48)	31.364	<0.001	b, c, d
Postprandial C-peptide (ng/mL)	4.60 ± 3.55	5.76 ± 3.55	7.73 ± 4.16	8.84 ± 6.04	6.53 ± 3.70	4.191	0.003	c
HOMA-IR	0.39 (0.28–0.81)	0.92 (0.55–1.31)	0.78 (0.55–1.48)	1.37 (0.80–1.92)	1.31 (0.71–2.76)	30.318	<0.001	a, c, d
HOMA-BETA	28.51 (18.22–43.93)	28.42 (15.58–39.25)	40.71 (30.44–71.99)	40.21 (19.75–61.17)	20.46 (10.84–46.72)	9.942	0.041	i
BETA/IR	52.10 ± 24.68	25.85 ± 2.87	52.14 (43.16–58.78)	28.52 (23.64–34.99)	15.59 (10.43–20.84)	30.318	<0.001	a, c, d, e, g, h, i
ΔI_120_/ΔG_120_	4.58 (1.47–7.59)	7.78 (−4.11–29.83)	9.56 (5.34–12.76)	10.53 (5.81–18.71)	2.35 (1.02–5.08)	20.733	<0.001	i, j
ΔI_120_/ΔG_120_/IR	9.51 (3.21–17.00)	11.15 (−3.47–20.54)	10.58 (6.08–16.19)	8.04 (3.64–15.94)	1.86 (0.81–3.36)	26.967	<0.001	d, g, i, j
ANGPTL8 (ng/mL)	0.38 ± 0.25	1.07 ± 0.52	0.92 ± 0.57	1.23 ± 0.48	0.85 ± 0.67	25.974	<0.001	a, b, c, d

M: male; F: female; BMI: body mass index; SBP: systolic blood pressure; DBP: diastolic blood pressure; TG: triglyceride; TC: total cholesterol; HDL-C: high-density lipoprotein cholesterol; LDL-C: low-density lipoprotein cholesterol; FINS: fasting serum insulin; PINS: postprandial serum insulin; HOMA-IR: homoeostasis model assessment of insulin resistance; HOMA-BETA: homoeostasis model assessment of beta cell function index. Pairwise comparisons: a, NGT versus IFG; b, NGT versus IGT; c, NGT versus CGI; d, NGT versus T2DM; e, IFG versus IGT; f, IFG versus CGI; g, IFG versus T2DM; h, IGT versus CGI; i, IGT versus T2DM; and j, CGI versus T2DM.

**(a):** 

	Model	Age		BMI		Waist circumferences		WHR		SBP	
		*r*	*P*	*r*	*P*	*r*	*P*	*r*	*P*	*r*	*P*
ANGPTL8	1	0.067	0.453	0.139	0.117	0.191	0.031	0.195	0.027	0.112	0.208
	2	—	—	—	—	0.132	0.142	0.140	0.120	0.118	0.188
Fasting C-peptide	1	−0.115	0.199	0.387	0.000	0.404	0.000	0.251	0.004	0.047	0.600
	2	—	—	—	—	0.174	0.052	0.096	0.287	−0.029	0.751

**(b):** 

	Model	DBP		TC		TG		LDL-C		HDL-C	
		*r*	*P*	*r*	*P*	*r*	*P*	*r*	*P*	*r*	*P*
ANGPTL8	1	−0.014	0.872	0.035	0.692	0.093	0.296	0.058	0.512	0.041	0.648
	2	−0.040	0.659	0.014	0.875	−0.087	0.335	0.039	0.662	0.039	0.669
Fasting C-peptide	1	0.140	0.117	0.163	0.067	0.401	0.000	−0.013	0.884	0.016	0.862
	2	0.010	0.913	0.147	0.102	0.152	0.090	−0.012	0.893	0.025	0.781

**(c):** 

	Model	HBA1C		FPG		PPG		FINS	
		*r*	*P*	*r*	*P*	*r*	*P*	*r*	*P*
ANGPTL8	1	−0.094	0.290	0.253	0.004	0.017	0.846	0.334	0.000
	2	0.052	0.567	0.198	0.027	0.066	0.468	0.116	0.197
Fasting C-peptide	1	0.011	0.898	0.241	0.006	0.215	0.015	0.881	0.000
	2	0.035	0.699	0.176	0.049	0.138	0.126	0.829	0.000

**(d):** 

	Model	Fasting C-peptide		PINS		Postprandial C-peptide		HOMA-IR	
		*r*	*P*	*r*	*P*	*r*	*P*	*r*	*P*
ANGPTL8	1	0.218	0.013	0.271	0.002	0.196	0.026	0.355	0.000
	2	0.204	0.022	0.093	0.305	0.177	0.049	0.135	0.134
Fasting C-peptide	1	—	—	0.701	0.000	0.665	0.000	0.874	0.000
	2	—	—	0.389	0.000	0.642	0.000	0.781	0.000

**(e):** 

	Model	HOMA-BETA		BETA/IR		ΔI_120_/ΔG_120_		ΔI_120_/ΔG_120_/IR	
		*r*	*P*	*r*	*P*	*r*	*P*	*r*	*P*
ANGPTL8	1	0.140	0.113	−0.252	0.004	0.127	0.153	−0.063	0.478
	2	0.033	0.713	−0.275	0.002	−0.023	0.799	−0.059	0.514
Fasting C-peptide	1	0.673	0.000	−0.240	0.006	0.331	0.000	−0.119	0.182
	2	0.605	0.000	−0.299	0.001	0.141	0.117	0.012	0.894

Model 2: adjusted for age and BMI. — means the analysis was not performed.

**Table 3:** Correlations between serum ANGPTL8 levels and lipids in NGT, IGR, and T2DM, respectively.

	TG		TC		HDL-C		LDL-C	
	*r*	*P*	*r*	*P*	*r*	*P*	*r*	*P*
NGT	−0.010	0.966	0.029	0.898	−0.049	0.829	0.048	0.830
IGR	−0.073	0.534	−0.108	0.362	−0.019	0.871	−0.071	0.550
T2DM	0.033	0.856	0.031	0.865	0.042	0.815	0.194	0.279
